# Replication Stress in Cancer: Mechanistic Insights and Therapeutic Opportunities for Radiosensitization

**DOI:** 10.3390/cimb48010067

**Published:** 2026-01-07

**Authors:** Spyridon N. Vasilopoulos, Ioanna Tremi, Ioly Kotta-Loizou, Angeliki Gkikoudi, Ourania E. Tsitsilonis, Sophia Havaki, Alexandros G. Georgakilas

**Affiliations:** 1 DNA Damage Laboratory, Physics Department, School of Applied Mathematical and Physical Sciences, National Technical University of Athens (NTUA), Zografou Campus, 15780 Athens, Greece; svasilopoulos@mail.ntua.gr (S.N.V.); ioannatre@med.uoa.gr (I.T.); angelikigkikoudi@mail.ntua.gr (A.G.); 2 Molecular Carcinogenesis Group, Department of Histology and Embryology, School of Medicine, National and Kapodistrian University of Athens, 11527 Athens, Greece; shavaki@med.uoa.gr; 3 Department of Life Sciences, Faculty of Natural Sciences, Imperial College London, London SW7 2AZ, UK; i.kotta-loizou13@imperial.ac.uk; 4 Section of Animal and Human Physiology, Department of Biology, School of Science, National and Kapodistrian University of Athens (NKUA), 15784 Athens, Greece; rtsitsil@biol.uoa.gr

**Keywords:** replication stress (RS), DNA damage response (DDR), radiosensitization, radiotherapy, ATR, Chk1, Wee1, PARP, RPA, oncogenes

## Abstract

Replication stress (RS) is a hallmark of cancer, largely driven by oncogene activation. Due to high levels of RS, cancer cells depend heavily on the RS response mechanisms to avoid DNA damage. This dependency creates a therapeutic opportunity that can be exploited for more effective cancer treatment. This review synthesizes current mechanistic understanding of RS and RS response and further describes how targeted disruption of RS response proteins (ATR, Chk1, Wee1, PARP, RPA) has been used in preclinical and clinical studies. We summarize preclinical and emerging clinical evidence for exploiting RS for radiosensitization, and outline candidate biomarkers and functional assays for patient selection. We also highlight the links between RS, therapy-induced senescence and innate immune activation via the cGAS–STING (cyclic GMP-AMP synthase—Stimulator of Interferon Genes) pathway, and address current challenges and future directions.

## 1. Replication Stress and Replication Stress Response

DNA replication is a central biological process that ensures the faithful transmission of the genetic information in the two daughter cells upon cell division. In eukaryotic cells, genomic DNA is replicated only once per cell cycle, in the S phase of interphase, and this process is vital for the growth, development, and maintenance of organisms [1,2]. DNA replication initiates from numerous genomic loci called replication origins. At these sites, the replisome, a complex of multiple proteins containing DNA helicase and DNA polymerase, unwinds the template DNA and synthesizes new DNA on both leading and lagging strands, creating the replication forks. Replication termination is marked by the convergence of replication forks, the disassembly of the replisome, and the resolution of the newly formed DNA molecules [3,4].

The DNA replication machinery encounters many challenges, which can ultimately lead to disruptions in replication fork progression, decreased replication fidelity, and even lethal DNA double-strand breaks (DSBs). Replication stress (RS) is a term that refers to the challenges and obstacles that emerge during the process of DNA replication, causing replication forks to slow or stall. RS is a complex phenomenon, for which there is neither a standard definition nor a specific set of cellular markers to characterize it [5,6]. Various causes, both intracellular and extracellular, can induce RS [7]. To summarize current knowledge, endogenous sources of RS include difficult-to-replicate DNA sequences, abasic sites, misincorporation of ribonucleotides into replicating DNA, DNA-protein crosslinks, replication–transcription conflicts, DNA secondary structures, oncogene activation [8], and nucleotide pool imbalances [9,10,11,12,13,14,15]. Exogenous sources include ultraviolet (UV) and ionizing radiation, as well as DNA-damaging agents, such as alkylating agents, crosslinking agents, and topoisomerase inhibitors [15,16,17]. These stressors trigger cellular responses to stabilize forks and address the DNA damage. The DNA damage response (DDR) and the RS response signalling pathways share many common proteins, and this overlap is important for preventing genomic instability [18,19].

The various factors that pose obstacles to fork progression and cause RS are traditionally believed to act directly *cis* at replication forks. However, recent findings propose that RS may also manifest independently of active forks, indicating a potential *trans-acting* role [20]. Additionally, disruptions in DNA repair processes and their products may impede replication, underscoring the diverse sources of RS [7,21,22].

When replication forks stall, e.g., in the presence of DNA damage, the mini-chromosome maintenance (MCM) replicative helicase continues to unwind the parental DNA downstream, revealing single-stranded DNA (ssDNA) [7]. This ssDNA is coated by Replication Protein A (RPA) and represents the signal for the RS Response. Ataxia telangiectasia and Rad3-related (ATR) kinase is recruited to RPA-ssDNA via the ATR-interacting protein (ATRIP) and is activated by the ATR activator Topoisomerase Binding Protein 1 (TOPBP1) [7,23]. Upon activation, ATR phosphorylates a variety of substrates, including checkpoint kinase 1 (Chk1) and histone H2AX. ATR-mediated checkpoint responses stabilize stalled forks and prevent entry into mitosis in the presence of damaged DNA [21,23,24]. However, when RS is enhanced, e.g., in the absence of ATR, and the amount of ssDNA exceeds the amount of RPA, RPA is depleted, leading to fork collapse and generation of DSBs [25]. Subsequent entrance to mitosis with non-replicated damaged DNA causes cell death through mitotic catastrophe. It is worth noting that this only occurs at very high levels of RS, whereas low to mild levels of RS cause genomic instability in cancer cells [26,27,28].

Although the RS response and DDR pathways share several core components, including ATR and Chk1, their outputs are highly context dependent. Functional specificity is primarily dictated by the nature of the initiating lesion and the phase of the cell cycle. In the S phase, when forks stall in the presence of DNA damage, ssDNA coated by RPA acts as the dominant signaling platform triggering the activation of ATR and its downstream effector Chk1. Accumulating evidence shows that ATR is a multifaceted regulator of replication fork stability via a variety of mechanisms: it suppresses new late origin firing to prevent RPA exhaustion, it prevents fork collapse by regulating fork reversal and nuclease activity, stabilizes the replisome, and facilitates fork restart and repair [29]. In contrast, once replication forks collapse and secondary DSBs are generated, signaling shifts towards the activation of Ataxia-telangiectasia mutated (ATM) kinase. The same also applies in the case of DSBs that are directly induced by ionizing radiation.

Despite the fact that the majority of DNA damage is repaired by the DDR pathways, it is likely that some unrepaired lesions can still remain in the DNA template during replication. To address this issue, cells activate the DNA damage tolerance (DDT) pathways. These complex signalling pathways allow DNA replication to proceed, despite the presence of lesions that could otherwise result in replication fork collapse, postponing the need for lesion repair [21,30,31].

This review focuses on the molecular origins of RS in cancer, its impact on DNA damage responses and radiosensitivity, and current therapeutic efforts to exploit replication-stress vulnerabilities—particularly through inhibition of ATR, Chk1, Wee1, and PARP. To define the scope of this review, we performed a structured literature search in PubMed/MEDLINE and ClinicalTrials.gov (for trials) to identify mechanistic, preclinical, and clinical studies relevant to RS, the RS response, and radiosensitization. Search terms included combinations of “*replication stress*”, “*replication stress response*”, “*ATR inhibition*”, “*Wee1 inhibition*”, “*Chk1 inhibition*”, “*RPA inhibitor*”, “*fork protection*”, “*radiosensitization*”, “*DDR inhibitors*”, “*oncogene-induced replication stress*”, and “*clinical trial*”. Publications up to November 2025 were considered. Priority was given to primary research articles that elucidate mechanistic links between RS and DDR, as well as translational and clinical studies evaluating DDR inhibitors and RS response-targeting agents alone or in combination with radiotherapy. Reviews were used for background and cross-referencing. Studies were included based on mechanistic relevance, experimental rigor, and translational importance; non-English language papers and case reports were excluded. Additional key references were identified through manual screening of reference lists from recent high-impact review articles. ClinicalTrials.gov entries were used to compile ongoing clinical studies (retrieval date: 30 November 2025), including information on trial phase, status, and available outcomes.

RS in cancer is increasingly recognized not simply as a static feature imposed by oncogenes, but as a dynamic vulnerability that can be therapeutically exploited. Recent studies show that inhibition of key RS response kinases such as ATR, Chk1, or Wee1—which cancer cells depend on to survive under RS—can tip the balance toward mitotic catastrophe, especially when combined with genotoxic therapy or in tumors with high intrinsic RS [32,33]. In the following sections, we will focus on the oncogene-related sources of RS in cancer, and we will explore the potential of targeting proteins of the RS response as a means to increase the sensitivity of cancer cells to radiotherapy.

## 2. Replication Stress in Cancer: The Role of Oncogenes

Cancer cells show elevated levels of RS in comparison to normal cells, and this contributes to their genomic instability [8,34,35]. A major cause for that is the activation of oncogenes. Overexpression or persistent activation of oncogenes, such as *CCNE1* (Cyclin E1) and *CCNE2* (Cyclin E2), oncogenic *HRAS*, and *c-Myc*, can cause RS by increasing the levels of replication origin firing [36,37]. The various molecular mechanisms of how oncogenes induce replication stress have been extensively reviewed by other groups [8,21,38,39]. In this section, we will summarize the most recent knowledge related to oncogene-induced RS, providing up-to-date data, with an emphasis on key oncogenes.

Cyclin E (Cyclin E1 and Cyclin E2) belongs to the cyclin family of proteins. Cyclin E directly binds to cyclin-dependent kinase 2 (CDK2) and the Cyclin E/CDK 2 complex stimulates the G1-S transition through the retinoblastoma (Rb)—E2F transcription factor pathway [40,41]. Aberrant Cyclin E expression or function has been observed in many cancers. Overexpression of Cyclin E has been found to shorten G1 and force cells into the S phase with an insufficient pool of nucleotides, leading to RS and genomic instability [42]. Two proposed mechanisms behind Cyclin E-induced RS in earlier studies were the increased origin firing and increased replication–transcription encounters. Increased origin firing, meaning many active replication forks, can lead to nucleotide depletion, and, changes in the origin firing density could interfere with the spatial separation of replication and transcription [37,43]. Recent work has shown that Cyclin E can induce RS via a variety of molecular mechanisms: interference with pre-replication complex (pre-RC) assembly, interference with origin licensing, disturbed origin firing, nucleotide pool depletion due to increased origin firing, and replication–transcription conflicts [44,45].

In a similar context, cell division cycle 25 A (Cdc25A—a phosphatase that activates CDK2) overexpression has been linked to accelerated G1-S transition and genomic instability resulting from RS [46,47,48]. Further evidence to support the role of these oncogenes in RS is provided by Neelsen et al., showing that overexpression of Cyclin E and Cdc25A induces replication fork slowing and replication fork reversal [49]. The finding of replication fork slowing is in line with the nucleotide depletion that was shown by Bester et al. [42].

In addition, oncogene activation can directly interfere with the deoxyribonucleotide (dNTP) biosynthesis pathway, causing depletion of the nucleotides that are necessary for DNA replication and therefore posing an obstacle to the progression of replication forks [50]. Bcl2, a major anti-apoptotic protein, causes replication stress in a similar way—by inhibiting the action of ribonucleotide reductase, an enzyme needed for the biosynthesis of dNTPs [51]. These data, in combination with the aforementioned findings, suggest that oncogenes can cause depletion of the dNTP pool that is necessary for DNA replication via two mechanisms: either by increased origin firing or by directly interfering with the dNTP biosynthesis pathway.

*c-Myc* is another oncogene that has been linked to RS. Activation of *c-Myc* has been linked to RS and DNA damage, by compromising the DDR pathways and by inducing the production of reactive oxygen species (ROS) [52]. c-Myc has also been found to interact directly with pre-RC components and replication origins [37]. Furthermore, G1-phase shortening by overexpression of Cyclin E and c-Myc, forces entry to S-phase before completion of transcription. This allows origin firing within transcribing genes, which, in turn, leads to replication–transcription conflicts, replication fork collapse, and DSB formation [53]. A recent study by Peripolli et al. proposed a novel mechanism for *c-Myc*-induced RS. According to this study, an increase in cohesins load on the DNA specifically at CCCTC-binding factor (CTCF) sites, driven by increased MAU2 levels, interferes with the progression of replication forks and causes RS [54].

The *RAS* family of genes (*HRAS*, *KRAS*, and *NRAS*) encode small GTPases that regulate cell proliferation and survival in response to extracellular signals [55]. Oncogenic *HRAS^G12V^* has been found to have a negative effect on DNA replication via a variety of mechanisms. It can cause increased origin firing leading to nucleotide depletion, as it has been observed for other oncogenes, it can directly interfere with ribonucleotide reductase, causing nucleotide depletion, and it can also lead to transcription-replication conflicts [39,56]. *HRAS^G12V^* overexpression in human fibroblasts (BJ fibroblasts) was shown to cause increased oxidative stress, resulting in slowed fork progression [57]. These data build on previous findings, where *RAS*-induced senescence was linked to increased ROS production. Furthermore, a recent study showed that oncogenic *KRAS^G12V^* induces RS, and this is attributed to chromatin compaction mediated by H3K27me3 (tri-methylation of lysine 27 on histone H3) and HP1 (heterochromatin protein 1). Interestingly, cells that survived *KRAS^G12V^*-induced RS exhibited increased ATR expression to ensure replication fork progression under the RS conditions. This ATR-mediated RS tolerance is dependent on Human primase and DNA-directed polymerase (PrimPol), and, while enabling cells to survive the RS, it also contributes to their genomic instability [58]. Recent work underscores that oncogene-driven RS is multifaceted. For example, cancers driven by mutant *RAS* not only show increased origin firing and nucleotide depletion, but also elevated R-loop formation and transcription-replication conflicts, leading to persistent fork stress and DNA damage. Such stress often engages the fork-protection machinery and checkpoint kinases (ATR/Chk1), making these tumors particularly dependent on RS response pathways and thus more vulnerable to RS-targeting inhibitors [59].

It is worth noting that such data highlight an interesting trade-off between cell survival and genomic instability during the early stages of tumorigenesis. Oncogene activation induces RS [8,60,61]. This oncogene-induced RS triggers an initial response, including DDR activation and oncogene-induced senescence (OIS), that acts as a barrier to tumor development. Some cells, however, acquire RS tolerance mechanisms that allow replication to continue. Such mechanisms may introduce errors, such as the PrimPol-dependent priming in the case of *KRAS^G12V^*-induced RS, thus promoting genomic instability. This small subset of cells, characterized by increased genomic instability, begins to clonally expand and represent the origin of cancer [58,62,63]. Therefore, while RS tolerance enables cell survival in the face of oncogene-induced RS, it comes at the cost of increased genomic instability, ultimately fuelling the development and progression of cancer.

As mentioned before, unusual DNA secondary structures, like stem-loops and G4-quadruplexes, are a cause of RS. These structures can be observed in common fragile sites, and it has been shown that RS in preneoplastic lesions is frequent within these regions [64].

Although *CCNE1*, *c-Myc*, and oncogenic *RAS* have well-established roles in elevating RS in cancer, they do so through partially distinct mechanisms that help explain tumor-specific vulnerabilities. Cyclin E overexpression primarily drives RS by shortening the G1 phase, excessive origin firing, dNTP depletion, and replication–transcription conflicts. *c-Myc*, apart from interference with origin firing dynamics and replication–transcription conflicts, additionally promotes ROS production and alters chromatin organization, including cohesin accumulation at CTCF sites, which collectively impair fork progression. In addition to these mechanisms, oncogenic *RAS* variants further expand the mechanistic landscape of RS by also causing chromatin compaction and engaging tolerance pathways such as PrimPol-mediated repriming. Notably, *c-Myc* overexpression has been shown to trigger RS rapidly, before any bioenergetic or metabolic changes in the cell, whereas *RAS* triggers RS with a delayed onset. In fact, the eventual slowing of replication forks in *RAS*-overexpressing cells coincides with bioenergetic metabolic changes and increased production of ROS [57]. Despite these mechanistic differences, all oncogenic contexts result in elevated RS and enforce a shared dependence on the RS response pathway, particularly the ATR/Chk1/Wee1 axis. More specifically, common downstream features include excessive ssDNA exposure, replication fork collapse or aberrant fork processing, checkpoint kinase activation, and high reliance on DDR and RS response pathways. This convergence provides a strong rationale for targeting RS response proteins across molecularly heterogeneous tumors.

Consistent with this context, a recent mRNA expression study by Guerrero Llobet et al. in 2022 showed that *CDC25A*, *CCNE1*, and *MYC* oncogenes overexpression induces RS in cancer cell lines. Further data derived from cancer patients revealed more amplified oncogenes that trigger replication stress, including, apart from the ones found in cell lines, *CCND1*, *MYB*, *MOS*, *ERBB2,* and *E2F1*. The study concluded with a signature of six genes, *AT10*, *DDX27*, *ZNF48*, *C8ORF33*, *MOCS3*, and *MPP6*, which are upregulated in response to oncogene-induced RS. This research provided a very important data pool for investigating the levels of RS in various cancer types [65].

## 3. Radiotherapy and Cancer Cell Radioresistance

Cancer remains one of the most important challenges in modern medicine and a leading cause of death worldwide. According to the most recent data from the World Health Organization (WHO)/International Agency for Research on Cancer, there were approximately 10 million cancer-related deaths in 2020 globally, and a significant increase in cancer cases worldwide is expected over the next twenty years.

Radiotherapy is a common cancer treatment modality that uses ionizing radiation to kill cancer cells. It is used either alone or often in combination with other therapies, like surgery and chemotherapy. Approximately 50% of cancer patients will receive radiotherapy during the course of their disease [66,67]. Radiotherapy contributes to long-term survival in almost half of cancer patients when combined with other treatment modalities [68]. Radiotherapy is currently used in the treatment of a wide range of solid tumors, including glioblastoma, lung, breast, prostate, colorectal, cervical, esophageal, and head and neck cancers [66,69].

Radioresistance, the development of cancer cell resistance to radiation treatment, is a major challenge to the effectiveness of radiotherapy, contributing to disease progression and poor patient prognosis. Radioresistance can be intrinsic or acquired. Intrinsic radioresistance is present before treatment, while acquired develops during or after radiotherapy [70].

Current studies have shed light on the mechanisms behind radioresistance. The ability of cancer cells to repair the DNA damage caused by radiation is the key factor. In addition, epigenetic mechanisms, like DNA methylation, histone modifications, and non-coding RNAs have been shown to play a role in the development of radioresistance [71,72].

DNA damage from the ionizing radiation used in radiotherapy can be either direct or indirect. In the former type of damage, radiation causes DNA lesions, such as single-strand breaks (SSBs) and DSBs. In the latter type of damage, the absorption of ionizing radiation by the water molecules in the cells generates ROS that cause oxidative damage [73]. DSBs are considered the most lethal type of DNA damage [74]. Actually, it has been shown that ionizing radiation is capable of causing clustered DNA damage—clusters of DNA lesions, all in close proximity, including SSBs, DSBs, oxidized base lesions, as well as regular and oxidized abasic sites [75,76,77]. This complex damage is the major cause of the genomic instability observed in irradiated cancer cells, reduces the effectiveness of the repair mechanisms, and causes cell death [78,79].

Cancer cells, like normal cells, respond to DNA damage caused by ionizing radiation through the DNA damage response and repair (DDR/R) signaling pathways. Enhanced DDR, and thus a higher DNA repair ability, has been observed in radioresistant cancer cells compared to normal cells [80]. This has also been observed in cancer stem-like cells (also referred to as cancer stem cells), a unique subset of tumor cells that have been isolated from leukemias and many types of solid tumors, including glioblastoma, breast cancer, prostate cancer, and melanoma [81,82,83]. Apart from enhanced DDR and cancer stem cells, hypoxia has been identified as another factor that contributes to radioresistance [84].

Based on all the above, targeting components of the DDR/R pathways is considered to be an attractive strategy for improving the effectiveness of chemotherapy and radiotherapy in cancer treatment. The combination of DDR inhibitors with immunotherapy and radiotherapy shows promising results regarding patient prognosis [85,86,87].

## 4. Targeting Replication Stress for Cancer Treatment and Radiosensitization

Cancer cells depend more on the RS response for their survival than normal cells [21,88]. Enhancing RS by targeting components of the RS response cascades has emerged as a promising approach in cancer therapy, to enhance the genotoxic and cytotoxic effects of chemotherapy and radiotherapy. Thus, small molecule inhibitors of the RS response and DDR cascades can act as radiosensitizers—molecules that potentiate the effectiveness of radiotherapy. In this section, we will summarize the current knowledge and status regarding molecular inhibitors that target major players in the RS response and DDR pathways.

### 4.1. Targeting ATR/Chk1, the Major Replication Stress Response Mediators

The activation of ATR/Chk1 is crucial for cancer cells to tolerate the enhanced replicative stress and avoid deleterious events such as mitotic catastrophe [89]. Schisandrin B, a natural product, was the first identified potential ATR inhibitor (ATRi), but its clinical application was restricted because of the requirement of a very high dose [90]. A few years later, VE-821, a selective ATRi, was shown to sensitize pancreatic cancer cells to the effects of radiotherapy in vitro [91]. It is worth noting that VE-821 was shown to be an effective radiosensitizer of radioresistant hypoxic tumor cells [92].

AZD6738 (also referred to as ceralasertib) is another potent and specific ATRi. It is orally active and developed by AstraZeneca [93]. The combination of AZD6738 with cisplatin improves the efficacy of cisplatin in ATM-deficient non-small cell lung cancer cells [94]. Furthermore, in a recent phase 1 study, combination of AZD6738 with carboplatin was shown to be well tolerated and presented a moderate clinical benefit for the participating patients [95]. ATRi AZD6738 has also gained particular attention due to its potential to act synergistically with radiotherapy and immunotherapy and improve antitumor immune responses. Vendetti et al. demonstrated that this ATRi enhances the cytotoxic effects of radiation and increases the CD8+ T cell activity in mouse models of *KRAS*-mutant cancer [96]. In accordance with these results, Sheng et al. also demonstrated that AZD6738 in combination with radiation increases the percentage of tumor-infiltrating CD8+ T cells and reduces the percentage of regulatory T cells in the tumor microenvironment. Triple treatment with AZD6738, radiation, and anti—programmed death-ligand 1 (anti-PD-L1) activated the cGAS-STING signalling pathway and demonstrated promising therapeutic potential in mouse models—it significantly inhibited tumor growth, prolonged survival, and delayed tumor recurrence [97]. Recent preclinical data also show that inhibition of ATR by AZD6738 radiosensitizes prostate cancer cells [98]. Furthermore, a recently published report of the first clinical study combining Ceralasertib with palliative radiotherapy showed that this combination was well tolerated and induced durable responses in patients with solid tumors [99].

Berzosertib (formerly M6620, VX-970, or VE-822) is another highly potent and selective ATRi [100]. Berzosertib has shown promising results in phase 1 clinical trials (NCT02157792, completed in 2020) in patients with advanced solid tumors [101]. Similarly to AZD6738, a recent study by Liu et al. showed that berzosertib enhanced the effects of radiotherapy and immunotherapy, by activating the cGAS-STING pathway and increasing the CD8+ T cell infiltration in mouse colorectal cancer models [102].

The aforementioned studies point to an immunomodulating effect of ATR inhibition in tumors. In accordance, the combination of radiation with AZD6738 or berzosertib has been shown to increase the release of High Mobility Group Box 1 (HMGB1) and the secretion of ATP, which are hallmark factors of immunogenic cell death, in human lung cancer and osteosarcoma cell lines [103]. Further supporting this effect, the combination of ablative radiotherapy with berzosertib was shown to activate the cGAS-STING pathway in lung cancer mouse models, and when combined with anti-PD-L1 it induced immunogenic cell death [104].

Another ATRi, elimusertib (BAY 1895344), has been reported to have a strong anti-tumor activity in lymphomas and to combine well with clinically approved PI3K inhibitor copanlisib in vitro and in vivo [105]. Furthermore, recent preclinical data support the radiosensitizing effect of this ATRi in HPV-negative head and neck squamous cell carcinoma (HNSCC) and in triple negative breast cancer cells [106,107].

Tuvusertib (M1774) is a potent oral ATRi recently evaluated as monotherapy in a first-in-human phase 1 trial in patients with advanced solid tumors, demonstrating manageable safety and exposure-related target engagement. The study supports tuvusertib’s further development and highlights biomarker-guided patient selection and combination strategies [108].

Chk1 is another important target for targeting the RS response. The phosphorylation and subsequent inactivation of Cdc25 by Chk1 is involved in S and G2/M checkpoint controls [109]. Furthermore, inhibition of Chk1 resulted in increased DNA replication initiation, an abnormal increase in ssDNA, and DSBs. These findings support a proposed model in which Chk1 is required for normal S-phase progression by minimizing aberrant initiation of DNA replication [110]. The inhibition of origin firing by Chk1 provides the cell with the necessary time for the resolution of the stress source [93].

The first Chk1 inhibitor that was developed in the 1990s, UCN-01, showed promising preclinical results, but in phase I trials, exhibited limited bioavailability due to high affinity with plasma α1-acid glycoprotein and severe side effects when the dose increased to overcome the bioavailability issue. Since then, more specific inhibitors of Chk1 have been developed. AZD7762 is such an example. It was found to radiosensitize p53 mutant cancer cells, promoting radiation-induced apoptosis in vitro and in xenografts [111,112]. LY2606368 (Prexasertib) and MK-8776 are other Chk1 inhibitors that have been tested in clinical trials, but they have shown limited clinical benefit and occasionally adverse side effects [33,113,114]. In a recent clinical trial (NCT03495323), the combination of Prexasertib with PD-L1 blockade was tolerable and demonstrated preliminary antitumor activity and evidence of cytotoxic T-cell activation in the peripheral blood of patients with high-grade serous ovarian cancer [115]. Furthermore, targeting the ATR/Chk1 axis with a CHK1 inhibitor was shown to significantly sensitize AT-rich interactive domain-containing protein 1A (ARID1A)-deficient colorectal tumors to radiotherapy by triggering cancer cell-intrinsic innate immunity through the cGAS-STING pathway [116].

To date, more than 15 Chk1 inhibitors have progressed into clinical trials, yet none has received marketing authorization [117]. It is worth noting that the severe side effects observed with Chk1 inhibitors reflect the broad role that Chk1 has in the cells, underlining the role of ATR/Chk1 signaling for genome integrity. In a 2025 study, Rawling et al. disclosed BEN-28010, a freely brain-penetrant, potent, and selective Chk1 inhibitor that, when combined with radiation, improved survival in mouse glioblastoma models [118].

### 4.2. Other Targets

#### 4.2.1. Wee1

Wee1 is a serine/threonine kinase that triggers G2/M arrest by inhibiting CDK1, under conditions of DNA damage. Thus, Wee1 inhibition can induce premature entrance to mitosis without adequate DNA repair [119]. Furthermore, Wee1 inhibition increases origin firing, leading to nucleotide depletion and RS [120,121]. These observations provide the rationale for using Wee1 as a target in cancer treatment: the inhibitors enhance RS in the cells and drive these cells into mitosis, resulting in cell death from mitotic catastrophe.

One such inhibitor is AZD1775 (development code of adavosertib). It has been tested in combination with chemotherapy or radiation, with promising antitumor activity [88]. AZD1775 demonstrated a radiosensitizing ability in various studies [88]. It was shown to radiosensitize hepatocellular carcinoma cells to protons and X-rays, independently of the *TP53* status of the cells. The mechanism for that is the induction of RS, as it was proven by the addition of nucleotides that reversed the radiosensitizing effect [122]. Similar results were also observed in esophageal cancer cells [123]. The combination of AZD1775 with chemotherapy and immunotherapy is also under clinical and preclinical investigation [124]. AZD1775 and anti-programmed cell death protein 1 (anti-PD-1) therapy effectively radiosensitize human and mouse hepatoma cells [125].

Recent studies have identified Wee1 as a potential promising therapeutic target in many cancer types characterized by defective checkpoint control. Wee1 inhibition by adavosertib and azenosertib (ZN-c3) selectively induced DNA damage and mitotic catastrophe in *ARID1A*-deficient, osimertinib-resistant EGFR-mutant non-small cell lung cancer models, while sparing normal fibroblasts, supporting Wee1 targeting as a promising strategy to overcome therapy resistance [126]. Similar results were obtained from a study in *ARID1A* and *TP53* dual-deficient colorectal cancer models, where Wee1 inhibition by MK-1775 or ZN-c3 resulted in pronounced DNA damage and cancer cell death [127]. ATIP3-deficient breast cancer cells are also highly sensitive to Wee1 inhibition, leading to mitotic catastrophe [128]. Importantly, Wee1 targeting has also demonstrated strong radiosensitizing potential. Wee1 inhibition by adavosertib leads to the radiosensitization of glioblastoma cells that are positive for EGFRvIII (epidermal growth factor receptor gene with deletions of exons 2–7), as it was shown in a recent study [129]. It is also worth noting that in HNSCC models, the combined inhibition of Chk1 and Wee1 markedly enhanced radiosensitivity to both X-rays and proton beam therapy [130]. Such results support Chk1/Wee1 inhibition as a promising strategy to sensitize tumors to radiotherapy. The importance of the combination of Wee1 inhibitors with ATR and Chk1 inhibitors is also pointed out by the fact that Wee1 upregulation has been implicated in the development of resistance to ATR and Chk1 inhibitors [131]. Currently, several Wee1 inhibitors are under development because of promising preclinical data [132].

#### 4.2.2. PARP

Poly (ADP-ribose) polymerases (PARPs) are nuclear proteins involved in DDR and they recruit DNA repair proteins to the sites of DSBs. PARP1 also controls fork speed and the choice of RS response mechanisms [133]. PARP inhibition causes RS-induced DNA damage, by preventing replication fork restart [87,88]. Another important characteristic of PARP inhibitors is that they are synthetically lethal in cancer cells with *BRCA* (Breast Cancer genes) mutations [33,134,135]. Four PARP inhibitors, olaparib, rucaparib, niraparib, and talazoparib, have been approved by the U.S. Food and Drug Administration (FDA) and by the European Medicines Agency (EMA). Olaparib was first approved in 2014 as maintenance therapy for platinum-sensitive advanced ovarian cancer with *BRCA1/2* germline mutations [136]. In 2020, the FDA approved olaparib and rucaparib to treat metastatic, castration-resistant prostate cancer, and olaparib was also approved for the treatment of pancreatic cancer [33]. Furthermore, olaparib has been reported to have strong radiosensitizing properties, which have been shown in clinical trials and in cholangiocarcinoma cell lines [137,138]. Emerging evidence indicates that combining RS response inhibitors (ATR and Chk1 inhibitors) with PARP inhibitors can overcome resistance, even in homologous recombination (HR)-proficient tumors. This combination exploits persistent RS and fork protection defects, expanding the therapeutic potential beyond classical *BRCA*-mutant cancers [139,140]. Furthermore, the combination of PAPR and Wee1 inhibition by olaparib and adavosertib respectively, induces effective radiosensitization of HPV-negative HNSCC cells [141]. A recent study presented a novel series of ATR/PARP1 dual inhibitors, developed by the fusion of AZD6738 and olaparib. One of them, B8, exhibits potent enzymatic activity and synergistic antitumor efficacy in triple-negative breast cancer models [142].

#### 4.2.3. RPA

RPA represents a potential therapeutic target in cancer treatment as it has been shown by some recent studies. HAMNO ((1Z)-1-[(2-hydroxyanilino)methylidene] naphthalen-2-one), TDRL-505, and fumaropimaric acid (NSC15520) are small molecule inhibitors that have been demonstrated to target the RPA70 subunit of RPA in vitro. HAMNO is the most studied one. It has been reported to increase the radiosensitivity of glioblastoma cancer stem-like cells, a very important observation, given that cancer stem-like cells are often radioresistant [143]. A recent study reported no significant changes in the survival of HAMNO-treated irradiated lung carcinoma cells [144], whereas Feng et al. demonstrated that RPA inhibition by HAMNO increased the radiosensitivity of nasopharyngeal carcinoma cells, an important result because radiotherapy is a standard therapeutic approach for nasopharyngeal carcinoma [145]. Our group has used an in silico drug design approach and designed a chemical compound with drug-like properties, targeting the RPA2 subunit and MLH1 [85].

In Table 1, we summarize all current, active clinical trials testing the efficacy and dose schemes of ATR, Chk1, Wee1, and PARP inhibitors.

Further supporting the mechanistic link between RS response inhibition and radiosensitization, Wong et al. showed that ATM, ATR, and PARP inhibitors induce markedly greater radiosensitization under fractionated versus single-dose irradiation in breast cancer cells, underscoring the need to test combination strategies in fractionated settings that mirror clinical practice [146].

Beyond the clinically advanced targeting of ATR, Chk1, Wee1, and PARP, which were discussed above, several emerging regulators of RS response and checkpoint control are gaining attention as potential therapeutic targets. For example, TOPBP1, the principal ATR activator, represents an attractive target to selectively disrupt RS responses upstream of ATR. A recent study identified a small-molecule inhibitor targeting TOPBP1, which disrupts TOPBP1 interactions with key partners, exerts antitumor activity, and synergizes with PARP inhibition in preclinical models [147]. MUS81, a nuclease that cuts stalled replication forks to allow restart or repair, also represents a promising target that has not been tested in clinical practice yet [33]. Currently, no selective small molecule MUS81 inhibitors are available, but it has been shown that MUS81-deficient gastric cancer cells are more vulnerable to Wee1 inhibition combined with immune checkpoint blockade therapy [148]. Components of DNA damage tolerance pathways, including RAD18, and the repriming enzyme PrimPol are also emerging targets. Recent evidence indicates that RAD18 contributes to the radioresistance of tumors by enhancing DSB repair [149]. Efforts focus on disrupting the interaction between RAD18 and its stabilizing partner, Melanoma Antigen A4 (MAGE-A4), which is often overexpressed in tumors [150]. This represents a promising approach to destabilize RAD18 and enhance the effects of radiotherapy and chemotherapy. The Ubiquitin-specific protease 1 (USP1) that participates in post-translational modifications of RS response proteins is currently receiving attention as a novel therapeutic target [131]. KSQ-4279 (RO7623066), a selective inhibitor of USP1, has been shown to overcome PARP inhibitor resistance in BRCA-mutant tumors [151]. This inhibitor has been tested in a phase 1 clinical trial (NCT05240898) with promising results. Other USP1 inhibitors, such as SIM0501, XL309–101, and HSK39775, are currently in early phases of clinical development [152]. In parallel, Membrane-associated tyrosine/threonine-protein kinase 1 (PKMYT1, MYT1), a G2/M checkpoint kinase, has recently emerged as a vulnerability in tumors with high levels of RS. When cells face replication stress, MYT1 works alongside Wee1 and phosphorylates CDK1, thus inhibiting it and creating a checkpoint to allow time for repair. Lunresertib is a first-in-class, orally available MYT1 inhibitor that is currently under clinical evaluation [153], (NCT06107868, NCT05605509). Although most of these targets remain at the preclinical or early clinical stage, their mechanistic roles in cellular response to RS provide a strong rationale for combination strategies with radiotherapy or established DDR inhibitors to amplify replication-associated DNA damage and overcome therapy resistance.

## 5. Replication Stress Biomarkers for Radiosensitivity Prediction

Proteins involved in the RS response pathways represent strong candidates for biomarkers because RS is a commonly observed feature across many cancer types. RS manifests as both molecular and functional defects, which can be quantified using a variety of assays. A pilot study using immunohistochemistry (IHC) on 32 archival formalin-fixed, paraffin-embedded (FFPE) tumor samples (from colon, lung, breast, and stomach cancers) analyzed the expression patterns of five proteins: Ki-67, Cyclin E, POLD3, γH2AX, and FANCD2, concluding that markers widely used in cell line studies require very careful evaluation before being utilized as useful biomarkers in clinical biopsy samples [154]. The accurate identification of tumors with high levels of RS or impaired DDR and RS responses is essential for selecting patients who may benefit from RS inhibition strategies, in combination with radiotherapy (or chemotherapy).

In Table 2, we summarize established and emerging RS biomarkers that may be used to predict tumor response to RS-targeting therapies and radiosensitivity.

Because there is no single specific RS marker established, we propose that a combined approach detecting a panel of biomarkers (e.g., combining γH2AX, p-RPA2, and RS-related gene expression) may increase accuracy over single markers for predicting tumor dependency on RS response, radiosensitivity, and therapy response.

In addition to protein- and transcriptomics-based biomarkers, specific genomic alterations related to RS response, DDR, or compromised cell-cycle checkpoint control are emerging as powerful predictors of response to strategies targeting the RS response. For example, a recent study identified that alterations in STK11/LKB1 and/or KEAP1 enhance the antitumor activity of ATR inhibitors in non-small cell lung cancer in vitro and in vivo [164]. In addition, loss of ARID1A, a SWI/SNF chromatin-remodeling factor, leads to elevated RS and impairs the G2/M checkpoint [165], thus rendering ARID1A-deficient tumors selectively vulnerable to Wee1 inhibition. Likewise, *TP53* mutations, which disrupt the G1/S checkpoint, enforce dependency on the G2/M checkpoint and sensitize cancer cells to Wee1 and Chk1 inhibition [127,166]. Strong support for genotype-driven patient stratification is provided by recent work from Ng et al., who found that combining radiation with an ATR inhibitor creates a synergistic “synthetic cytotoxicity” specifically in ATM deficient tumors. Beyond ATM, the study identifies a broader panel of 20 genes, such as *MCPH1*, that sensitize cells to both ionizing radiation and ATR inhibitors [167]. Synthetic lethality between ATR inhibition and ATM deficiency has also been shown previously in gastric cancer cells and other tumors [131,168], as cells with loss of ATM rely only on ATR for orchestrating DDR.

Taken together, the aforementioned data advocate for a biomarker- and genotype-guided patient stratification in RS-targeting and radiosensitization strategies. Integrating genomic profiling with RS-associated protein markers and functional assays is likely to improve patient selection and maximize the therapeutic benefit of RS response inhibition combined with radiotherapy. Given that preclinical and clinical data in this field accumulate continuously, based on current knowledge and taking into account some of the most common genomic alterations in cancers, we propose that for tumors with ATM mutations, ATR inhibitors could be used as radiosensitizers, in tumors with p53 mutations ATR, Wee1, and Chk1 inhibitors could be used as radiosensitizers, and in tumors with *BRCA* mutations, PARP inhibitors can be prioritized as radiosensitizers.

## 6. Discussion and Future Perspectives

The interplay between the RS response and the DDR represents a promising research area for the development of novel cancer therapies. Accumulating research and emerging clinical data show that targeting the RS response and the DDR enhances cancer cell sensitivity to DNA damage caused by radiotherapy, primarily by destabilizing stalled replication forks and impairing checkpoint control. The shared components of these pathways, such as ATR and Chk1, have already been targeted in ongoing clinical trials, demonstrating the potential of this approach. It is also worth noting that novel approaches to cancer treatment, combining molecular inhibitors, radiotherapy, and immunotherapy, seem to hold great promise for the future of cancer treatment, taking advantage of their synergistic effects [87].

Cancer cells are relatively more sensitive to RS response and DDR inhibitors than normal cells. A major reason for this is that cancer cells are characterized by elevated intrinsic RS, and therefore, they rely more on the RS response pathway, compared to normal cells [21,88]. This relative tumor selectivity is further reinforced by the frequent defects in cell-cycle control and DDR signaling pathways that characterize cancer cells. For example, high reliance on ATR/Chk1 signaling and deficiencies in ATM, ARID1A, and BRCA1/2 render tumor cells hypersensitive to specific pharmacological inhibitors. In contrast, normal proliferating tissues typically retain intact cell cycle checkpoints and have lower baseline RS.

Intertumoral heterogeneity (between patients with the same tumor type) and intratumoral heterogeneity (within individual tumors) are hallmarks of cancer [169]. In the context of RS, this heterogeneity is reflected in differences in intrinsic RS levels, dependence on RS response pathways, DNA repair capacity, and sensitivity to DDR inhibition [170]. As a result, subclonal populations with distinct DDR defects, replication dynamics, and cell-cycle checkpoint dependencies, are expected to exist in tumors, resulting in heterogeneous responses to ATR, Chk1, Wee1, or PARP inhibition. In the context of radiotherapy, such heterogeneity is also relevant, as subclonal populations with reduced RS or enhanced repair mechanisms may preferentially survive combined treatments, promoting adaptive resistance. Integrating genomic profiling, biomarker-guided approaches, and adaptive treatment strategies may therefore be essential to maximize the antitumor efficacy while limiting resistance arising from tumor heterogeneity.

A major challenge in exploiting RS therapeutically is toxicity to normal proliferating tissues, which rely on intact ATR/Chk1 signaling for genome stability. Consequently, optimizing tolerability is a central challenge in clinical translation. Current knowledge of normal tissue toxicities associated with the use of RS response and DDR inhibitors derives largely from the clinical use of PARP inhibitors, with increasing data emerging for ATR, Chk1, and Wee1 inhibitors. The most commonly reported adverse effects mentioned are hematological and bone marrow toxicities, gastrointestinal disorders, and fatigue [171]. When combined with radiotherapy, the adverse off-target effects may intensify, considering the effects of ionizing radiation on healthy tissues. Furthermore, the combination of DDR inhibitors and chemotherapy has been reported to cause increased bone marrow toxicity, compared to either treatment alone [87]. Several strategies could help mitigate these limitations and improve the therapeutic window. At first, careful planning of dosage and scheduling schemes is required [172]. Short-course administration or intermittent dosing of the RS response inhibitors synchronized with fractions of radiotherapy may also be of benefit. Importantly, the interaction between radiotherapy schedule and inhibitor administration timing is critical, particularly in the context of fractionated radiotherapy, which remains the standard in modern clinical practice [146]. Notably, intermittent dosing and alternating regimens are two strategies that could also help overcome the issue of drug resistance, thus further improving therapeutic efficacy and limiting normal tissue toxicities [173]. Furthermore, sequential rather than fully concurrent treatment schedules may preserve the radiosensitizing effect while reducing damage to normal tissues. In addition, tumor-targeted delivery approaches, such as nanoparticle-based carriers [157], may enhance intratumoral drug accumulation while limiting systemic exposure. Patient selection in oncology is crucial in personalized medicine approaches. Biomarker-driven selection of patients with tumors exhibiting high levels of intrinsic RS can increase the likelihood of enhanced antitumor effects and benefits for the patients. Conversely, biomarkers associated with normal tissue radiosensitivity should also be considered during trial design to minimize the risk of adverse effects [174].

Another major challenge that is increasingly reported is the issue of drug resistance to RS response inhibitors. A well-studied example is resistance to PARP inhibitors, which arises through multiple mechanisms. These include restoration of homologous recombination via reversion mutations or re-expression of homologous recombination genes that were silenced, mutations in PARP1 that decrease PARP trapping, and increased drug efflux via upregulation of specific pumps [131,175]. These adaptive mechanisms can be counteracted by combining PARP inhibition with ATR, Chk1, or Wee1 inhibitors, or even epigenetic drugs [131,176,177]. The main identified mechanisms of resistance to ATR inhibitors are coming from preclinical studies and include loss of Cdc25A, mutations in forkhead box M1 (FOXM1) transcription factor, and mutations in epithelial cell transforming sequence 2 (ECT2) Rho GTPase exchange factor [175]. A novel mechanism identified in gastric cancer involves the loss of UPF2, a component of the nonsense-mediated mRNA decay pathway. Loss of UPF2 reduces transcription-replication collisions, meaning that the cell is less dependent on ATR-mediated fork recovery to survive [178]. Regarding Chk1 inhibitors, recent preclinical data point to Wee1 upregulation as a main mechanism of resistance [179]. In a recent study, Murray et al. showed that tumor-intrinsic PD-L1 determines resistance to Chk1 inhibitors, as PD-L1 depletion enhanced the efficacy of Chk1 inhibitors [180]. Combination and biomarker-based strategies represent an effective means to overcome resistance to RS response inhibitors.

Emerging evidence suggests that RS is not only a driver of genomic instability but can also trigger therapy-induced senescence (TIS) in cancer cells. TIS is a state of cellular senescence (growth arrest) in cancer cells triggered by radiotherapy or chemotherapy [181]. RS contributes to treatment resistance, as it has been shown for glioblastoma. In glioblastoma cell lines, targeting ATR reduced TIS, pushing these cells towards apoptosis. When combined with a PARP-1 inhibitor, ATR inhibition eliminated the senescent cell population even more effectively, highlighting a promising strategy: targeting RS not only to sensitize cells to radiation, but also to target TIS with the potential of better treatment outcomes [182]. Building on this, there is a translational rationale for combining senolytic therapies with RS response inhibition. In triple-negative breast cancer, the combination of PARP inhibition (talazoparib) and irradiation robustly induced senescence, and the subsequent treatment with the senolytic navitoclax (ABT-263) led to apoptosis of the senescent cells and reduced tumor regrowth in vivo [183]. Together, these studies suggest that pushing cells into a senescent state via RS-targeted therapy plus irradiation, and then treating them with senolytics, may have the potential to improve long-term outcomes by eliminating the persistent senescent population. Furthermore, combined targeting of RS and senescence can also be a promising approach. In various lymphoma models, the combination of ATR and PI3K inhibition, by treatment with elimusertib and copanlisib, respectively, has shown in vitro and in vivo antitumor activity [105].

Recent work also highlights how RS can be leveraged to modulate antitumor immunity, particularly through activation of the cGAS-STING pathway. Accumulating preclinical and early clinical data confirms that combining RS response inhibitors with immunotherapy, especially immune checkpoint inhibitors, represents a promising strategy that increases DNA damage and tumor immunogenicity. Cancer cells treated with RS response inhibitors may proceed to mitosis with damaged DNA, leading to cell death via mitotic catastrophe. DNA fragments generated following mitotic catastrophe and/or micronuclei formation are released in the cytoplasm and are sensed by the cGAS-STING pathway. The cGAS-STING pathway senses cytosolic dsDNA, triggering type I interferon signaling, dendritic cell activation, and promoting cytotoxic T-cell infiltration [184,185]. While this pathway is essential for immune recognition, chronic or low-level activation, observed often in tumors with high chromosomal instability and persistent DNA damage, creates an immunosuppressive environment by upregulating PD-L1 expression [186]. This explains the rationale of combining RS response-targeting therapy with anti-PD-L1 immune checkpoint inhibition therapy. In the same context, a recent study demonstrated that enhancing replication stress by Chk1 inhibition can be coupled with anti-PD-1 immunotherapy to improve antitumor responses. It is also noteworthy that the authors developed manganese-based nanoparticles carrying the Chk1 inhibitor PF477736 [187]. As mentioned before, such nanoparticle-based delivery platforms may help widen the therapeutic window by enhancing tumor-selective accumulation of DDR inhibitors, reducing systemic toxicity, and enabling rational multi-modality combinations. A promising multi-modal therapeutic strategy is to combine RS response or DDR inhibitors with radiotherapy and immunotherapy to overcome cancer’s ability to resist single-modality treatments [87]. Finally, it is worth mentioning that computational and “omics”-based approaches are a valuable help in the discovery of potential therapeutic targets in many diseases, including cancers [188,189]. Furthermore, data from these approaches can lead to the identification of new RS-associated targets and predictive biomarkers and also facilitate the design of new inhibitors with drug-like properties. The characterization of specific RS biomarkers can improve the selection of patients who may benefit from RS response-targeting strategies.

## 7. Concluding Remarks

High levels of RS represent a defining vulnerability of cancer cells that can be exploited to enhance the efficacy of radiotherapy. Targeting RS response pathways promotes replication fork collapse, increases DNA damage burden, and disrupts checkpoint control, thereby amplifying radiation-induced cytotoxicity. The combination of the G2/M checkpoint impairment caused by ATR/Chk1 and Wee1 inhibition, and the DNA damage caused by ionizing radiation, forces cells to enter mitosis prematurely, with unrepaired damaged DNA, leading to mitotic catastrophe. Beyond direct cytotoxic effects, strategies that target the RS response and enhance RS can also induce senescence and generate cytosolic DNA, linking DNA damage to innate immune activation through the cGAS-STING pathway. Emerging approaches combining DDR inhibition with senolytics, immunotherapy, or nanoparticle-based drug delivery offer promising opportunities to improve the therapeutic index and overcome the severe issue of resistance to therapy. Rational patient stratification using RS-associated biomarkers has the potential to increase future clinical success.

**Figure 1:**
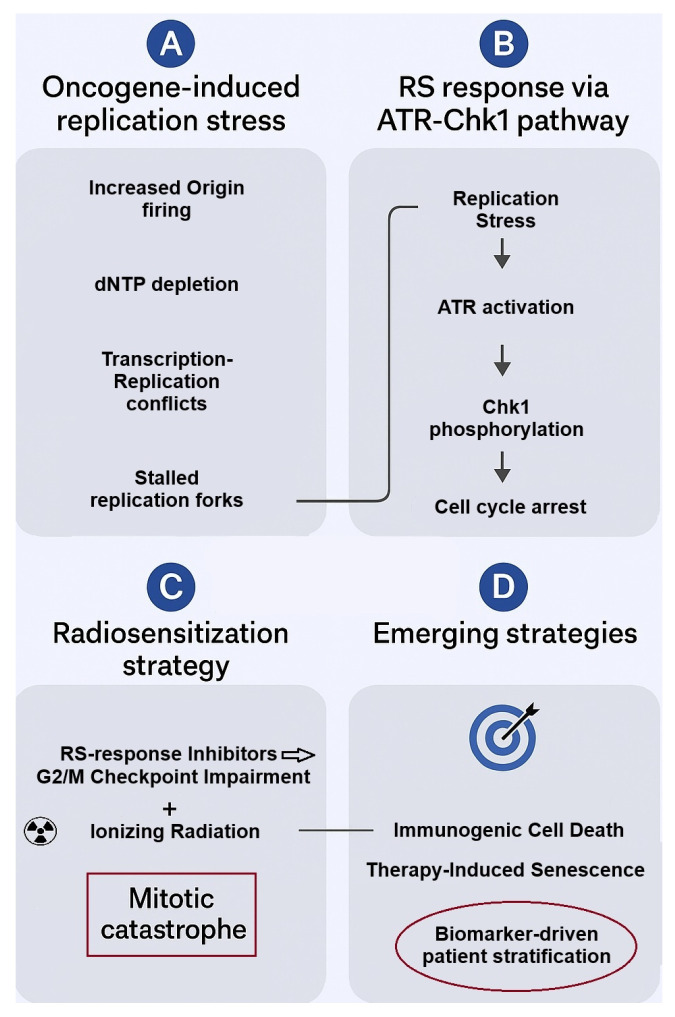
Schematic overview of replication stress (RS) in cancer cells, RS response, and therapeutic strategies for radiosensitization. (**A**) Oncogene activation induces replication stress, resulting in stalled replication forks. (**B**) The ATR/Chk1 pathway is activated in response to replication stress, promoting fork stabilization and cell cycle arrest to maintain genome integrity. (**C**) Therapeutic inhibition of RS response factors (ATR, Chk1, Wee1, PARP, RPA), combined with ionizing radiation, leads to replication fork collapse and mitotic catastrophe, enhancing radiosensitivity. (**D**) Emerging strategies: RS response inhibition combined with radiotherapy promotes immunogenic cell death and therapy-induced senescence (TIS), which can subsequently be exploited using senolytic agents. Specific biomarkers like the ones mentioned in this review can help identify patients who will most likely benefit from the aforementioned approaches.

Below, we provide a visual summary (Figure 1) of the major concepts covered in this review.

As many RS response inhibitors are currently under preclinical and clinical development, we expect that, in the near future, such strategies will offer benefits to cancer patients.

## Figures and Tables

**Table 1:** Current ongoing clinical trials of molecular inhibitors targeting the replication stress response in various cancers. Data retrieved from https://www.clinicaltrials.gov/. Retrieval date: 30 November 2025.

Target	Drug—Inhibitor	Active Combination Therapy/Type	Prior RT Requirement	Cancer Type	NCT Number	Phase	Status	Biomarker-Enriched Population
**Chk1 ^a^**	PEP07	Monotherapy	NO	Acute Myeloid Leukemia or Lymphoma, Mantle-Cell	NCT05659732	1b	Recruiting	Replication stress–high
	BBI-355	Monotherapy	NO	Tumors With Oncogene Amplifications	NCT05827614	1/2	Recruiting	Oncogene amplification–associated replication stress
		Erlotinib (TK Inhibitor)						
		Futibatinib (TK Inhibitor)						
		BBI-825 (RNR inhibitor)						
	PEP07	Monotherapy	NO	Advanced or Metastatic Solid Tumors	NCT05983523	1	Recruiting	Unselected (replication stress–oriented rationale)
	Prexasertib (ACR-368)	Monotherapy	NO	Endometrial Adenocarcinoma	NCT05548296	2	Recruiting	TP53-mutant (enriched)
		Gemcitabine/Chemotherapy						
**ATR ^b^**	Ceralasertib (AZD6738)	Monotherapy	NO	Chronic Lymphocytic Leukemia	NCT03328273	1	Active, not recruiting	ATM-deficient/DDR-altered
		Acalabrutinib/BTK Inhibitor						
	Berzosertib (M6620)	Gemcitabine/Chemotherapy	NO	Leiomyosarcoma, Adult	NCT04807816	2	Recruiting	HRD-enriched
	Elimusertib (BAY 1895344)	Monotherapy	NO	Relapsed or Refractory Solid Tumors	NCT05071209	1/2	Active, not recruiting	Unselected (exploratory DDR biomarkers)
	Berzosertib (M6620)	Gemcitabine Hydrochloride/Chemotherapy	NO	Recurrent Ovarian, Primary Peritoneal, or Fallopian Tube Cancer	NCT02595892	2	Active, not recruiting	BRCA/HRD-enriched
	AZD6738	Monotherapy	NO	Leukemia and Myelodysplastic Syndrome	NCT03770429	1	Recruiting	DDR-altered hematologic malignancies
	AZD6738	Olaparib/PARP Inhibitor	NO	Resistant Prostate Cancer	NCT03787680	2	Active, not recruiting	HRD/DDR-altered
	Elimusertib (BAY 1895344)	Gemcitabine/Chemotherapy	NO	Advanced Pancreatic and Ovarian Cancer, and Advanced Solid Tumors	NCT04616534	1	Active, not recruiting	HRD-enriched
	Berzosertib (M6620)	Lurbinectedin/Chemotherapy	NO	Small Cell Cancers and High-Grade Neuroendocrine Cancers	NCT04802174	1/2	Recruiting	Replication stress–high
	RP-3500	RP-6306/PKMYT1 Inhibitor	NO	Advanced Solid Tumor	NCT04855656	1	Recruiting	DDR-altered/replication stress–high
	Berzosertib (M6620)	Topotecan Hydrochloride/Chemotherapy	NO	Small Cell Lung Cancers and Small Cell Cancers Outside of the Lungs	NCT03896503	2	Recruiting	Replication stress–high
	AZD6738	Monotherapy	NO	Solid Tumor Refractory to Conventional Treatment	NCT02223923	1	Active, not recruiting	Unselected
		Palliative radiotherapy						
	Ceralasertib (AZD6738)	Monotherapy	NO	Solid Tumors	NCT03682289	2	Recruiting	RT-induced replication stress
		Olaparib/PARP Inhibitor						
		Durvalumab/Immunotherapy						
	Elimusertib (BAY 1895344)	Pembrolizumab/Immunotherapy + SBRT	NO	Recurrent Head and Neck Cancer	NCT04576091	1	Active, not recruiting	DDR-altered/RT-associated stress
	Elimusertib (BAY 1895344)	Cisplatin/Chemotherapy	NO	Advanced Solid Tumors with Emphasis on Urothelial Cancer	NCT04491942	1	Active, not recruiting	Platinum-sensitive/DDR-altered
		Cisplatin + Gemcitabine Hydrochloride/Chemotherapy						
	Tuvusertib (Μ1774)	Avelumab/Immunotherapy	YES	Endometrial Cancer	NCT06518564	2	Recruiting	DDR-altered
	Tuvusertib (M1774)	Lartesertib/ATM Inhibitor	NO	Metastatic or Locally Advanced Unresectable Solid Tumors	NCT05396833	1b	Active, not recruiting	ATM-deficient
		Avelumab/Immunotherapy						
	Tuvusertib (M1774)	Monotherapy	NO	Metastatic or Locally Advanced Unresectable Solid Tumors	NCT04170153	1	Active, not recruiting	Unselected (DDR exploratory)
		Niraparib/PARP inhibitor						
	Tuvusertib (M1774)	Niraparib/PARP Inhibitor	NO	Ovarian Cancer	NCT06433219	2	Recruiting	BRCA/HRD-enriched
		Lartesertib/ATM Inhibitor						
		Niraparib/PARP Inhibitor + Lartesertib/ATM Inhibitor						
**Wee1 ^c^**	Azenosertib (ZN-c3)	Monotherapy	NO	Platinum-Resistant High-Grade Serous Ovarian, Fallopian Tube or Primary Peritoneal Cancer	NCT05128825	2	Recruiting	TP53-mutant
	Debio0123	RP-6306/PKMYT1 Inhibitor	NO	Advanced Solid Tumor	NCT04855656	1	Recruiting	Replication stress–high
	Zentalis (ZN-c3)	Gemcitabine/Chemotherapy	YES	Pancreatic Cancer	NCT06015659	2	Recruiting	TP53-mutant/replication stress–high
	Adavosertib (AZD-1775 or MK-1775)	Radiation Therapy	NO	Incurable Esophageal and Gastroesophageal Junction Cancers	NCT04460937	1	Active, not recruiting	TP53-mutant/RT-associated stress
	Adavosertib (AZD-1775 or MK-1775)	Monotherapy	NO	Recurrent or Persistent Uterine Serous Carcinoma or Uterine Carcinosarcoma	NCT03668340	2	Active, not recruiting	TP53-mutant
	Adavosertib (AZD-1775 or MK-1775)	Monotherapy	NO	Cancers With BRCA Genetic Changes	NCT04439227	2	Active, not recruiting	BRCA-mutant/HRD
	Azenosertib (ZN-c3)	Carboplatin/Chemotherapy	NO	Ovarian Cancer	NCT04516447	1b	Recruiting	TP53-mutant/platinum-resistant
		Pegylated liposomal Doxorubicin Chemotherapy						
		Paclitaxel/Chemotherapy						
		Gemcitabine/Chemotherapy						
		Bevacizumab/Immunotherapy						
**PARP ^d^**	Olaparib	Pembrolizumab /Immunotherapy	NO	Advanced BRCA-mutated or HDR-defect Breast Cancer	NCT03025035	2	Active, not recruiting	BRCA/HRD
	Niraparib	Copanlisib (PI3K inhibitor)	NO	Recurrent Endometrial, Ovarian, Primary Peritoneal, or Fallopian Tube Cancer	NCT03586661	1	Active, not recruiting	PI3K-altered/HRD
	Niraparib	Ipilimumab/Immunotherapy	NO	Metastatic Pancreatic Adenocarcinoma	NCT06747845	2	Recruiting	DDR-altered
	Talazoparib	Tazemetostat/Epigenetic therapy	NO	Metastatic Castration-resistant Prostate Cancer	NCT04846478	1a/1b	Active, not recruiting	HRD/epigenetic dysregulation
	Niraparib	Dostarlimab/Immunotherapy + Radiation Therapy	YES	Triple Negative Breast Cancer	NCT04837209	2	Active, not recruiting	HRD-enriched
	Niraparib (Treatment Extension Study)	Monotherapy	NO	Ovarian and Breast Neoplasms	NCT04641247	2	Recruiting	BRCA/HRD
	Saruparib (AZD5305)	Monotherapy	NO	Advanced Solid Malignancies	NCT04644068	1/2	Active, not recruiting	Selective PARP1/HRD
		Paclitaxel/Chemotherapy						
		Paclitaxel, Carboplatin/Chemotherapy						
		T-Dxd/Antibody-Drug Conjugate						
		Dato-DXd/Antibody-Drug Conjugate						
		Camizestrant						
	Fluzoparib	Dalpiciclib (CDK4/6 inhibitor) + Endocrine therapy	NO	HR+/HER2-Advanced Breast Cancer	NCT05759546	2	Recruiting	HRD-exploratory
	Niraparib	Monotherapy	NO	Recurrent IDH 1/2 Gliomas	NCT05406700	Early 1	Active, not recruiting	HRD-independent
		Resection Surgery						
	Niraparib	Temodal/Chemotherapy	YES	Glioblastoma	NCT06258018	1/2	Not yet recruiting	DDR-altered

**Notes: a.** CHK1 inhibitors are mainly investigated in tumors with high replication stress and proliferative signaling. Proposed biomarkers include activation of replication stress pathways (e.g., p-CHK1, γH2AX), oncogene-driven replication stress (e.g., *MYC* or *CCNE1* amplification), and TP53 deficiency. Clinically, CHK1 inhibitors are explored primarily in hematologic malignancies and selected solid tumors, including endometrial, ovarian, and small cell lung cancers, often in combination with DNA-damaging chemotherapy. **b.** ATR inhibitors target tumors with elevated replication stress or defects in DNA damage response pathways. Identified biomarkers include replication stress signaling (e.g., p-ATR, γH2AX), oncogenic drivers of replication stress (e.g., *MYC* overexpression, *RAS* mutations), and loss of DDR components (e.g., *ATM*, *BRCA1/2*, *ARID1A*, *TP53*). ATR inhibitors have shown promise in non-small cell lung cancer (NSCLC), ovarian cancer, Triple-negative breast cancer (TNBC), urothelial carcinoma, pancreatic cancer, and prostate cancer and are frequently combined with DNA-damaging agents, PARP inhibitors, or radiotherapy. **c.** Wee1 inhibitors are preferentially explored in tumors lacking G1 checkpoint control, particularly those with *TP53* mutation or loss. Additional proposed biomarkers include high replication stress (e.g., γH2AX, p-RPA32), elevated CDK1/2 activity, *CCNE1* amplification, and defects in DNA repair pathways (e.g., BRCA1/2, RAD51, FANCD2). Clinical development has focused mainly on gynecologic malignancies, platinum-resistant high-grade serous ovarian cancer, and uterine serous carcinoma, and also on gastrointestinal and pancreatic cancers, as monotherapy or in combination with chemotherapy or radiotherapy. **d.** PARP inhibitors exploit synthetic lethality in tumors with homologous recombination deficiency (HRD). Established biomarkers include pathogenic alterations in *BRCA1/2* and other homologous recombination genes (e.g., *PALB2*, *RAD51*), as well as genomic HRD signatures. PARP inhibitors have demonstrated robust efficacy in biomarker-selected ovarian, breast, prostate, and pancreatic cancers and are increasingly evaluated in combination regimens. **Abbreviations: RT:** Radiotherapy; **TK:** Tyrosine Kinase; **RNR:** ribonucleotide reductase; **BTK:** Bruton’s tyrosine kinase; **SBRT:** Stereotactic Body Radiation Therapy; **T-Dxd:** trastuzumab deruxtecan; **Dato-DXd:** datopotamab deruxtecan; **HRD:** homologous recombination deficiency.

**Table 2:** RS biomarkers for tumor radiosensitivity prediction.

Biomarker	Biological Rationale	Prediction for DDR Inhibitor Sensitivity	Assay/Detection Method	Key References
γH2AX	Marker of DSBs and stalled or collapsed forks. Persistent γH2AX foci indicate defective repair and correlate with radiosensitivity.	High levels indicate sensitivity to ATR/Chk1/Wee1 inhibitors and combined radiotherapy.	Immunofluorescence or immunohistochemistry to detect nuclear foci. Flow cytometry.	[155]
53BP1 (p53-binding protein 1)	Involved in the repair of DSBs. Marker of unrepaired replication-associated lesions that are indicative of RS and replication fork instability.	Presence of 53BP1 foci indicates ongoing replication stress and potential response to ATR/Chk1 inhibitors.	Immunofluorescence to detect nuclear 53BP1 foci.	[156]
Phospho-RPA2	Reflects the activation of the ATR-dependent RS response and subsequent fork processing.	Predicts tumor response to chemotherapy and PARP inhibitors.	Immunofluorescence or Western blot.	[157,158]
ATR/Chk1 Axis Activation (p-ATR, p-CHK1)	Indicators of RS response activation. Markers of potential sensitivity to ATR/Chk1 axis inhibitors.	Active signaling indicates likely responsiveness to ATR and CHK1 inhibitors.	Immunofluorescence or immunohistochemistry.	[159,160]
dNTP pool imbalances	A known cause of RS. Leads to the activation of cGAS-STING pathway.	Imbalances indicate nucleotide depletion, predicting sensitivity to ATR, Chk1, and Wee1 inhibitors.	HPLC	[13,161]
Oncogene-induced RS gene signature (*CCNE1*, *CDC25A*, *MYC*)	Transcriptional signature of oncogene-induced RS.	High signature predicts replication stress dependency and potential benefit from ATR, Chk1, and Wee1 inhibitors.	RNA-seq	[65]
RAD51 foci (absence)	Lack of RAD51 foci after DNA damage indicates that HR is not functioning which indicates that DSB repair is impaired.	Absence predicts strong sensitivity to PARP inhibitors and potential sensitivity to ATR, Chk1 and Wee1 inhibitors.	Immunofluorescence	[162]
FANCD2	A marker of fork protection. Its loss indicates vulnerability to fork collapse.	Low levels predict sensitivity to PARP inhibitors and potential synergy with ATR inhibitors.	Immunofluorescence	[163]

## Data Availability

No new data were created or analyzed in this study. Data sharing is not applicable to this article.

## Abbreviations

The following abbreviations are used in this manuscript:

ARID1A	AT-rich interactive domain-containing protein 1A
ATM	Ataxia-telangiectasia mutated kinase
ATR	Ataxia telangiectasia and Rad3-related kinase
ATRi	ATR inhibitor
ATRIP	ATR-interacting protein
BRCA1/2	Breast cancer genes 1 and 2
Cdc25A	Cell division cycle 25 A
cGAS	Cyclic GMP–AMP synthase
Chk1	Checkpoint kinase 1
DDR	DNA damage response
DDR/R	DNA damage response and repair
DDT	DNA damage tolerance
dNTP	deoxyribonucleotide
DSBs	DNA double-strand breaks
ECT2	Epithelial cell transforming sequence 2
EGFR	Epidermal growth factor receptor
EMA	European Medicines Agency
FDA	Food and Drug Administration
FOXM1	Forkhead box M1 transcription factor
H3K27me3	tri-methylation of lysine 27 on histone H3
HMGB1	High Mobility Group Box 1
HNSCC	Head and neck squamous cell carcinoma
HP1	Heterochromatin protein 1
MAGE-A4	Melanoma Antigen A4
MCM	Mini-chromosome maintenance (replicative helicase)
MYT1	Membrane-associated tyrosine/threonine-protein kinase 1
OIS	Oncogene-induced senescence
PARP	Poly(ADP-ribose) polymerase
PD-1	Programmed cell death protein 1
PD-L1	Programmed death-ligand 1
PrimPol	Human primase and DNA-directed polymerase
ROS	Reactive oxygen species
RPA	Replication protein A
RS	Replication stress
SSBs	Single-strand breaks
ssDNA	Single-stranded DNA
STING	Stimulator of interferon genes
TIS	Therapy-induced senescence
TOPBP1	Topoisomerase-binding protein 1
USP1	Ubiquitin-specific protease 1
UV	Ultraviolet radiation
WHO	World Health Organization
